# Shoot Multiplication and Callus Induction of *Labisia pumila* var. *alata* as Influenced by Different Plant Growth Regulators Treatments and Its Polyphenolic Activities Compared with the Wild Plant

**DOI:** 10.3390/molecules26113229

**Published:** 2021-05-27

**Authors:** Mat Yunus Najhah, Hawa Z. E. Jaafar, Jaafar Juju Nakasha, Mansor Hakiman

**Affiliations:** 1 Department of Crop Science, Faculty of Agriculture, Universiti Putra Malaysia (UPM), Selangor 43400, Malaysia; najhahyunus@gmail.com (M.Y.N.); hawazej@upm.edu.my (H.Z.E.J.); jujunakasha@upm.edu.my (J.J.N.); 2 Laboratory of Sustainable Resources Management, Institute of Tropical Forestry and Forest Products, Universiti Putra Malaysia (UPM), Selangor 43400, Malaysia

**Keywords:** shoot multiplication, callus induction, plant growth regulator, antioxidant activities, polyphenolic activities, *Labisia pumila* var. *alata*

## Abstract

This study aims to investigate whether the in vitro-cultured *L. pumila* var. *alata* has higher antioxidant activity than its wild plant. An 8-week-old *L. pumila* var. *alata* nodal segment and leaf explants were cultured onto Murashige and Skoog (MS) medium supplemented with various cytokinins (zeatin, kinetin, and 6-benzylaminopurine (BAP)) for shoot multiplication and auxins (2,4-dichlorophenoxyacetic acid (2,4-D) and picloram) for callus induction, respectively. The results showed that 2 mg/L zeatin produced the optimal results for shoot and leaf development, and 0.5 mg/L 2,4-D produced the highest callus induction results (60%). After this, 0.5 mg/L 2,4-D was combined with 0.25 mg/L cytokinins and supplemented to the MS medium. The optimal results for callus induction (100%) with yellowish to greenish and compact texture were obtained using 0.5 mg/L 2,4-D combined with 0.25 mg/L zeatin. Leaves obtained from in vitro plantlets and wild plants as well as callus were extracted and analyzed for their antioxidant activities (DPPH and FRAP methods) and polyphenolic properties (total flavonoid and total phenolic content). When compared with leaf extracts of in vitro plantlets and wild plants of *L. pumila* var. *alata*, the callus extract displayed significantly higher antioxidant activities and total phenolic and flavonoid content. Hence, callus culture potentially can be adapted for antioxidant and polyphenolic production to satisfy pharmaceutical and nutraceutical needs while conserving wild *L. pumila* var. *alata.*

## 1. Introduction

*Labisia pumila* (Blume) Mez is a member of the Myrscinaceae family, and it is known by several vernacular names, such as Selusuh Fatimah, Tadah Matahari, Mata Pelanduk Rimba, and others [1]. It is primarily found in Southeast Asia’s lowlands and forests with hills [2], and Ibrahim and Jaafar [3] described it as a shade-loving plant that thrives in thinned jungle with 70% to 90% shade. This plant is well known as a medicinal plant, with a long history of use in traditional medicine, especially for postpartum medicine for pregnant women [4,5,6].

Previous studies have shown that, in addition to being a postpartum medicine, *Labisia pumila* also has biological activities, such as antioxidant, antimicrobial, antifungal, anti-inflammatory, anti-carcinogenic, anti-proliferative, and phytoestrogenic effects and can prevent post-menopausal osteoarthritis [7,8,9,10,11,12]. All these medicinal uses are due to the phytochemical contents of *L. pumila*, which include resorcinol, flavonoids, and phenolic acids [13,14], which have been proven by previous studies.

*L. pumila* has been sought after since the discovery of its medicinal properties, and there is a strong demand for its raw materials on the market [4]. However, because of its slow growth rate, the source of this herb in its natural habitat is diminishing [2]. As a result, plant tissue culture techniques are used to promote large-scale production in order to maximize supply and ensure that this plant does not become extinct. Callus initiation is the first step in cell growth and the development of secondary metabolites [15]. There have been a few studies on enhancing essential oils, antioxidant activity, and secondary metabolite production [16,17], particularly polyphenolics via callus culture [2,6,18], but none have been conducted on *L. pumila.*

Antioxidants are the chemical compounds that may postpone or slow the onset of a lipid oxidation reaction in the food system or substances that oppose or inhibit the oxidation reaction promoted by oxygen or peroxides [19], also known as reactive oxygen species (ROS) [20]. ROS oxidation can cause cell membrane disintegration, membrane protein damage, and DNA mutation, all of which can lead to aging and the development of other diseases [19]. As excess free radicals circulating in the body, they oxidize low-density lipoproteins (LDL) and cause other serious diseases [20].

Antioxidants’ primary role is to bind to free oxygen radicals and prevent these radicals from harming healthy cells. By breaking free radical chain reactions and sacrificing their electrons, the molecule may prevent another molecule from oxidizing [21,22]. In other words, antioxidants serve as free radical scavengers by reacting to reactive radicals and neutralizing them, resulting in a less dynamic, risky, and toxic substance than the radicals that have been eliminated. 

Phenolics are the largest group of secondary metabolites. They all have one or more phenol groups as standard features and they range in complexity from a simple aromatic ring structure to a highly complex polymeric structure [23], with the majority being water-soluble and biosynthetically derived from the shikimic acid pathway [24,25]. Phenolic compounds are colorless liquids that can produce flammable liquids that burn with a sooty flame due to incomplete combustion [21]. Moreover, phenolic compounds have structural features including a phenol ring backbone, in which a carbon atom is bound to a hydroxyl group (OH) or other substitutes such as sugar molecules and organic acids [24,26,27]. 

In several trials, callus extract was compared to in vivo plant parts for its antioxidant properties and polyphenolic content, and it was discovered that callus extract can have higher or lower antioxidant properties than its source explants [28]. Since there has been little research on the antioxidant and polyphenolics of *L. pumila* var. *alata*, especially on callogenesis, the current study examines the ability of its plantlets to multiply and induce callus under various plant growth regulators. The antioxidant and polyphenolic activities of wild plants, in vitro plantlets, and the callus of *L. pumila* var. *alata* were also compared. The findings point to a new way to produce antioxidants that do not require wild plant harvesting.

## 2. Results

### 2.1. Shoot Multiplication

**Figure 1:**
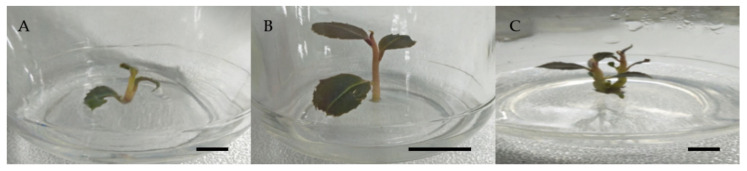
The effect of different concentrations of zeatin on growth of *Labisia pumila* var. *alata* compared to the control after 6 weeks of culture. (**A**) Stunted growth (4 mg/L zeatin), (**B**) healthy growth (2 mg/L zeatin), and (**C**) control treatment. Horizontal bar = 1 cm.

The present study showed that, after six weeks of culture, the number of shoots, length of shoots, and the number of leaves were all affected by different plant growth regulators at different concentrations for shoot multiplication of *L. pumila* var. *alata* using the nodal segment as the explant. The results of this study revealed major differences between three plant growth regulators—BAP, kinetin, and zeatin—with zeatin outperforming the other two hormones. In comparison to BAP and kinetin, MS supplemented with 3, 1, and 2 mg/L zeatin has shown the highest mean results, which are 6 to 7 shoots, 4 cm, and 3 to 4 in the number of shoots, length of shoots, and the number of leaves on each culture, respectively (Table 1, Figure 1).

According to Table 1, the highest mean in the number of shoots was observed in 2 and 3 mg/L zeatin, followed by 1 mg/L BAP, and the lowest in 5 mg/L kinetin. Furthermore, 1 mg/L zeatin also recorded the highest mean of length of shoot, followed by control, 1 mg/L BAP, and 2 mg/L zeatin. The kinetin concentration of 5 mg/L and the control treatment had the lowest mean of this parameter. Table 1 also revealed that 2 mg/L zeatin had the highest mean number of leaves, followed by control, 2 mg/L, and 3 mg/L zeatin, while 2 mg/L BAP recorded the lowest mean of this parameter.

### 2.2. Callus Induction

#### 2.2.1. Callus Induction Using a Single Concentration of 2,4-D and Picloram

**Figure 2:**
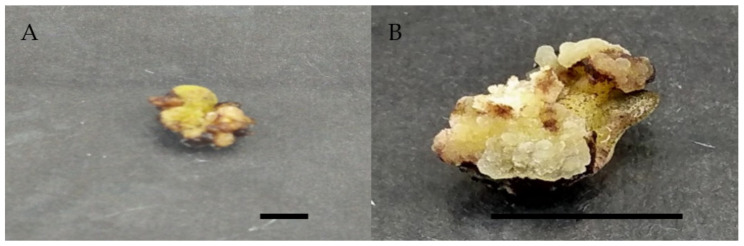
Callus induction of *Labisia pumila* var. *alata* on MS medium with single concentration of 2,4-D and picloram after 4 weeks of culture. (**A**) 0.5 mg/L 2,4-D and (**B**) 1.0 mg/L picloram. Horizontal bar = 1 cm.

For these two auxins, there are substantial variations in callus induction percentage, with 0.5 mg/L 2,4-D and 1.0 mg/L picloram having the highest mean percentage of callus induction. Table 2 shows that 0.5 mg/L 2,4-D had the highest callus induction rate of 60%, followed by 1 mg/L picloram with a 50% callus induction rate. The control treatment, which had no callus induced, had the lowest callus induction percentage. Other picloram concentrations, on the other hand, had a higher mean callus induction percentage than other 2,4-D concentrations. In the current study, all concentrations of picloram were able to induce more callus than other concentrations of 2,4-D, despite the fact that 0.5 mg/L 2,4-D showed the highest callus induction compared to 1 mg/L picloram (Table 2). In the current study, callus induced with 0.5 mg/L and 1 mg/L 2,4-D had a greenish and compact texture, while callus induced with 1.5 mg/L had a friable, yellowish to greenish callus texture (Figure 2).

#### 2.2.2. Callus Induction using Combination of 0.5 mg/L 2,4-D and 0.25 mg/L Different Cytokinins

Further investigation was carried out using a combination of 0.5 mg/L 2,4-D and 0.25 mg/L cytokinins (zeatin, kinetin, BAP, TDZ), with the results revealing a large difference between them. The combination of 0.5 mg/L of 2,4-D with 0.25 mg/L zeatin produced the highest callus induction percentage of 100%, followed by kinetin with 73.4% and TDZ with just 6.6% (Table 3).

**Figure 3:**
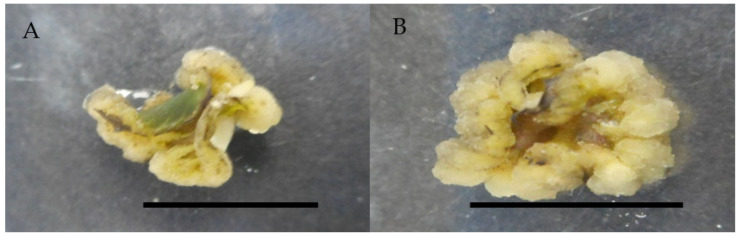
Callus induction of *Labisia pumila* var. *alata* on MS medium with combination of 2,4-D and cytokinins after 4 weeks of culture. (**A**) 0.5 mg/L 2,4-D + 0.25 mg/L zeatin and (**B**) 0.5 mg/L 2,4-D + 0.25 mg/L kinetin. Horizontal bar = 1 cm.

All combinations of 2,4-D with BAP, kinetin, and TDZ produced friable and yellowish to greenish callus in the current study, while zeatin produced compact and greenish callus (Table 3, Figure 3).

#### 2.2.3. Callus Induction Using a Combination of 1.0 mg/L Picloram and 0.5 mg/L Cytokinins

**Figure 4:**
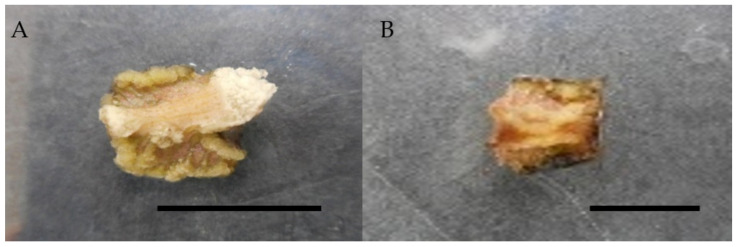
Callus induction of *Labisia pumila* var. *alata* on MS medium with the combination of picloram and cytokinins after 4 weeks of culture. (**A**) 1.0 mg/L picloram + 0.5 mg/L kinetin and (**B**) 1.0 mg/L picloram + 0.5 mg/L BAP. Horizontal bar = 1 cm.

The current study also discovered that combining 1 mg/L picloram with 0.5 mg/L kinetin resulted in the highest callus induction percentage, with 80%, followed by kinetin with 66.6%, and the lowest with just 20% callus induction with TDZ (Table 4). In the current study, picloram combined with BAP and picloram combined with kinetin induced callus that was whitish and compact and whitish and friable, respectively (Table 4, Figure 4). In the current research, a combination of 1 mg/L picloram, 0.5 mg/L TDZ, and zeatin induced compact, whitish to dark brown and compact, greenish callus, respectively.

### 2.3. Total Antioxidant, Phenolic, and Flavonoid Content in Wild Plant and In Vitro Culture of Labisia pumila var. alata

#### 2.3.1. Total Phenolic and Flavonoid Content in Wild Plant, In Vitro-Derived Plantlet, and Callus of *Labisia pumila* var. *alata*

The phenolic content of each extract differed significantly. The highest total phenolic content was recorded in callus extract (1.9 mg GAE/g DW), followed by the leaf extract of the in vitro plantlet (1.44 mg GAE/g DW) and the leaf extract of the wild plant (1.01 mg GAE/g DW) (Table 5). In comparison to leaf extracts from the wild plant (1.67 mg QE/g DW) and leaf extracts from in vitro plantlets (1.41 mg QE/g DW), total flavonoid content in the callus extract (2.38 mg QE/g DW) was significantly higher. 

#### 2.3.2. Total Antioxidant (DPPH and FRAP Methods) in Wild Plant, In Vitro-Derived Plantlet, and Callus of *Labisia pumila* var. *alata*

Table 6 shows the DPPH free radical scavenging activity of the three sample sources. The in vitro plantlet leaf extract (57.47 mg TE/g DW) and wild plant leaf extract (40.45 mg TE/g DW) had lower antioxidant activity than *L. pumila* var. *alata* callus extract (75.88 mg TE/g DW). Table 6 also showed that the in vitro callus from the leaf explant of *L. pumila* var. *alata* had the highest antioxidant activity (151.76 mg TE/g DW) using the FRAP method, followed by the leaf extract from the in vitro plantlet (115 mg TE/g DW) and the leaf extract from the wild plant (80.91 mg TE/g DW).

**Figure 5:**
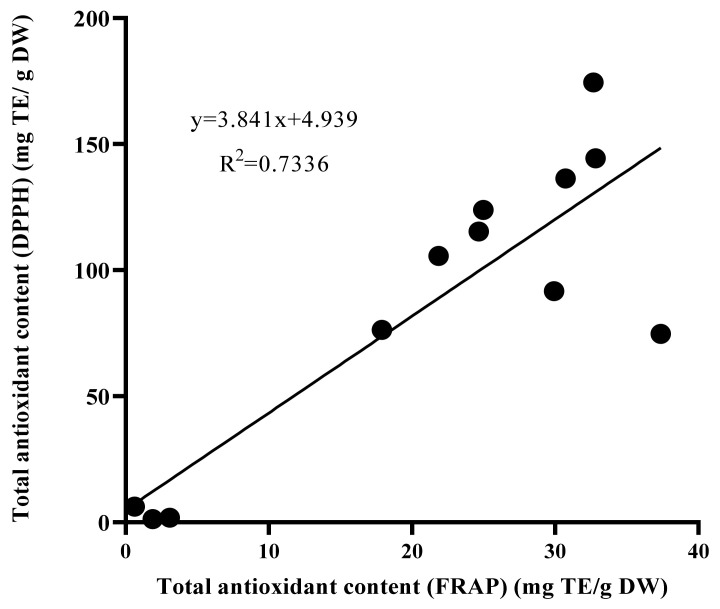
Correlation (R^2^) between antioxidant activities using FRAP and DPPH methods in leaf extract of wild plant, in vitro plantlet, and callus of *Labisia pumila* var. *alata*. DPPH = 2,2-diphenyl-1-picrylhydrazyl, FRAP = ferric reducing antioxidant potential.

The results of Pearson correlation analysis, shown in Figure 5, indicate a highly significant and positive linear correlation between the total antioxidant activities findings using the DPPH and FRAP methods (R^2^ = 0.7336).

#### 2.3.3. Correlation between Antioxidant Activities and Total Phenolic and Flavonoid Content in Wild Plant, In Vitro-Derived Plantlet, and Callus of *Labisia pumila* var. *alata*

The DPPH activity was significantly correlated with the FRAP activity in different extracts of *L. pumila* var. *alata*, as shown in Table 7. The same findings were observed for total phenolics and total flavonoids, with both polyphenolic contents being significantly and positively correlated with total antioxidant activities using both DPPH and FRAP methods.

## 3. Discussion

In some studies, tissue culture techniques were found to be more successful than conventional methods such as cutting techniques and seed germination in establishing *L. pumila* var. *alata* plantlets [14,29]. The current findings are consistent with those of Marbawi et al. [30], who discovered that, using the nodal segment as an explant, the optimal concentration and type of plant growth regulator for shoot multiplication is 20 µM zeatin. In addition, 1–7 mg/L of zeatin induced up to 100% shoot regeneration in *L. pumila* var. *alata*, according to Ling et al. [4]. 

Tekdal and Cetiner [31] discovered that zeatin was superior to other cytokinins during *Thermopsis turcica* Kit Tan et al. shoot regeneration. In *Solanum nigrum* L. and *Vaccinium corymbosum* L., zeatin has also been linked the development of shoots [32,33]. The stimulating effect of zeatin during shoot regeneration may be attributed to the lower affinity of cytokinin-degrading enzyme for zeatin, allowing zeatin to be comparatively higher in concentration and thus having a higher potential for organogenesis in plants than any other cytokinin at the same concentration [34,35].

The present research, on the other hand, found significant differences in the shoot multiplication responses of *L. pumila* var. *alata* plantlets with BAP treatment versus kinetin treatment in *L. pumila* var. *alata* plantlets. The optimum BAP concentration for inducing shoots and leaves, according to Table 1, is 1 mg/L. In a study by Nisha Rani and Nair [36], BA was found to be more successful than kinetin in a shoot regeneration study, which is consistent with the findings of Sahoo and Chand [37] and Chandramu et al. [38]. *Withania somnifera* (L.) Dunal nodal explants treated on MS medium supplemented with 1 mg/L BAP yielded the highest number of shoots (8.0). Studies on medicinal and aromatic plant species such as *Psoralea corylifolia* L. [39], *Eclipta alba* (L.) Hassk. [40], and *Mentha viridis* L. [41] also proved that BAP is superior to other cytokinins for shoot regeneration in these species.

As shown in Figure 1, raising the concentration of plant growth regulators until it reached 3 mg/L increased the shoot multiplication response, but it decreased once it exceeded 3 mg/L. This finding is in line with studies on *Mentha viridis* L. [41], *Scrophulana kakudensis* Franch [42], *Psoralea* *corylifolia* L. [39], and *Terminalia arjuna* Roxb. [43] cultures, which found that the number of shoots per culture gradually increased when increasing the cytokinin concentration from 1.0 to 3.0 mg/L of BAP, with shoot proliferation declining after 3 mg/L.

Furthermore, 2,4-D is the most significant PGR used for the proliferation of callus, callus induction, and also callus maintenance, according to Rao et al. [44]. *Michelia champaca* (Magnoliaceae) Linn callus may also be induced on MS medium supplemented with 2,4-D at a concentration of 2 mg/L or lower, according to Abdelmaged et al. [45]. A 100% callus induction was observed in the current study when a combination of 0.5 mg/L 2,4-D and 0.25 mg/L zeatin was used. Callus induction can occur when there is an intermediate ratio and interaction of exogenous auxin and cytokinin, causing cellular differentiation and organogenesis in tissue and organ cultures [30,46].

The combination of zeatin and NAA induced undesirable hard callus formation within 6 weeks of culture initiation, resulting in indirect organogenesis and delay in shoot regeneration [30]. The combination of 0.4 mg/L 2,4-D and 1.6 mg/L zeatin promoted callus induction of the seeds of flax plant [47], while 100% callus induction was also found on *Hibiscus syriacus* L. leaf explants when MS medium was supplemented with 0.5 mg/L 2,4-D and 0.5 mg/L zeatin [48]. When leaf explants were supplemented with a combination of 1 mg/L picloram and 0.5 mg/L BAP, up to 67% callus induction was observed in the current study. In their research, Oi et al. [49] discovered that medium containing 3 mg/L picloram, 2 mg/L glycine, 1000 mg/L glutamine, and 0.05 mg/L BAP was the best for pineapple (*Ananas comosus* (L.) Merr.) callus induction.

For antioxidant analysis in *L. pumila* var. *alata*, leaves from the wild plant, in vitro plantlet, and callus were used as samples. The DPPH free radical scavenging method and the ferric reducing antioxidant potential (FRAP) method were used to measure total antioxidants [50,51]. These findings indicate that in vitro propagated callus and leaves have a greater capacity to scavenge the DPPH radicals and reducing ferric ions. Compared to the in vitro plantlet and leaf from the wild plant, the callus extract showed a nearly two-fold and one-fold increase in scavenging capacity and in reducing ferric ions, respectively.

According to a previous analysis, the leaf callus has a higher total phenolic, flavonoid, and squalene content than wild explants, resulting in the highest antioxidant activity (IC_50_ = 88.8 mg/mL) using the DPPH assay [52]. In vitro methanol stem extracts and ethanol leaf extracts had the highest antioxidant properties, with IC_50_ values of 0.248 ± 0.45 mg/mL and 0.397 ± 0.67 mg/mL, respectively, while in vivo chloroform stem extracts had lower antioxidant activity (IC_50_ of 10.99 ± 0.24 mg/mL) [53]. Mint, tapioca, Indonesian bay-leaf, sweet potato, ‘cekur manis’, and betel leaves are other plants that have been assessed using this method and showed a strong capability of reducing ferric ions. Three spices, which are basil, laurel, and juniper, also display ferric ion reducing activity [54].

Previous studies on *Labisia pumila* [2,6,8,18] have reported that it has been identified to contain a few phytochemicals. Most of them are phenolic compounds, including phenolic acids and flavonoids, and other detectable constituents have been shown to have high antioxidant properties spectrophotometrically by testing their free radical scavenging, ferric reducing antioxidant power (FRAP), and Β-carotene bleaching activities using extracts from wild plants but none from extracts of in vitro cultured plants. Studies also found that leaves from all wild plant varieties displayed higher antioxidant activities compared to stem and roots. 

The current study also found that the total phenolic content in the callus extract was significantly higher than that in the in vitro plantlets and wild samples. In contrast to wild-grown plants, the callus extract in methanol solvent had the highest phenol content [55]. The higher phenolic content in callus extracts suggests that in vitro callus cultures, rather than field-grown plant organs, may produce high-concentration bioactive compounds with antioxidant activity [56]. The total flavonoid content results also recorded the same pattern of the total phenolic content, except for the total flavonoid content of in vitro plantlets, which not significantly different from the wild plant extracts. Mustapha and Harun [57] found a similar pattern of findings when comparing the total flavonoid content in callus and wild leaf extracts, demonstrating that the callus tissue has the same phytochemicals as the mother plant, showing that plant cells grown in culture have the ability to synthesize bioactive compounds that are identical to the parent plant from which they are generated.

Callus cultures are usually favored over intact plants for secondary metabolite development because callus in vitro growth is not affected by seasonal or climatic conditions, allowing it to be accessible all year [28]. Though intact plant organs produce secondary metabolites, cultures of undifferentiated cells (calli) are a rich source of a wide range of secondary metabolites [56,58]. The superiority of callus in producing the highest antioxidant activity compared to other sources of the extract is due to its totipotency and plant growth hormone treatment (55) and most likely due to a lack of morphological differentiation in callus cultures, which is often necessary for higher secondary metabolite development [59].

The existence of antioxidants in *L. pumila* var. *alata* was attributed to these identified compounds, as demonstrated by the positive correlation between DPPH and FRAP assays with total phenolic content and total flavonoid content in the present study. These positive correlations mirrored those found in Manivannan et al.’s [42] study, which found a strong positive correlation between the total phenol, flavonoid content, and antioxidant content of tissue extracts. As cancer prevention agents and also for other human disorders, phenolic compounds and flavonoids are applied to the vast majority of organic compounds in higher plants, including medicinal and officinal plants [60]. A positive correlation between antioxidant activity and phenolic content has also been reported in other studies [32,61,62].

The reasons that in vitro cultured plants and callus recorded higher content of antioxidants might be related to plant growth regulators and nutrient content in the cultured medium causing oxidative stress or regulation in the tested plants, thus increasing antioxidant enzymes such as CAT, SOD, POD, and APX [63,64,65]. Plant growth regulators such as cytokines and auxins may have induced polyphenol synthesis during in vitro cultivation [55]. Since callus growth in a nutrient-rich culture medium is inevitably subjected to greater carbon influx than field-grown plant specimens, it may affect the metabolic flux for the biosynthesis of elevated levels of phenolics [66]. The production of phenolics in the callus is determined by the abundance of the carbon source in the medium [67], as well as the growth rates of the cultured tissue [68] and the levels of auxin/cytokinin in the medium [69,70].

## 4. Materials and Methods

### 4.1. Plant Materials and Maintenance

In vitro plantlets of *Labisia pumila* var. *alata* were obtained from Forest Research Institute of Malaysia (FRIM), Kuala Lumpur, Malaysia and propagated onto MS [71] supplemented with 1 mg/L BAP (*w*/*v*), 3% (*w*/*v*) sucrose, and 3 mg/L Gelrite (*w*/*v*) at pH 5.7. The plantlets were maintained for 8 weeks and subcultured at 4-week intervals before the subsequent experiment. 

### 4.2. Media Preparation

MS solidified medium contained 0.1 g/L *myo*-inositol, 0.0001 g/L thiamine-HCL, 0.0005 g/L pyridoxine-HCL, 0.0005 g/L nicotinic acid, 0.5 g/L casein hydrolysate, 0.5 g/L proline, and 30 g/L sucrose and was solidified with 3 g/L Gelrite. The pH of this medium was adjusted to pH 5.7 before autoclaving. For the shoot multiplication experiment, the PGRs used were zeatin, kinetin, and BAP, and for each PGR, six different concentrations were used: 0 (control), 1, 2, 3, 4, and 5 mg/L for each PGR. 

In the callus induction experiment, the PGRs used were 2,4-D (0.5 mg/L) and picloram at six different concentrations (0 (control), 0.5, 1.0, 1.5, 2.0 and 2.5 mg/L). Later, the optimum concentration of 0.5 mg/L 2,4-D and 1 mg/L picloram was combined with 0.25 mg/L and 0.5 mg/L different cytokinins.

### 4.3. Shoot Multiplication

Eight-week-old stem-containing nodes from in vitro plantlets were used as the explants for the shoot multiplication experiments. The stem was cut into 1-1.5 cm sections, each with a node, and cultured on hormone-free MS medium (MSO) and MS basal media supplemented with different cytokinin concentrations. The number and length of shoots formed and the number of leaves formed were examined after 6 weeks of culture. Shoot length was measured as the height of the shoot.

### 4.4. Callus Induction and Maintenance

The leaves of 8-week-old in vitro plantlets were excised and used as explants for callus induction. The leaf was excised into 4 mm × 4 mm pieces with sterile scalpels and cultured on hormone-free MS medium (control) and MS basal medium combined with various concentrations of auxins alone or in combination with various concentrations of cytokinins. After 4 weeks of culture, the data callus score, percentage of callus formation, morphology, and texture of the callus were all collected.

### 4.5. Measurement of Total Antioxidant Activity

#### 4.5.1. Extract Preparation

First, 0.5 g fully expanded leaves from the wild plant of *L. pumila* var. *alata* grown for 6 months, leaves from in vitro plantlets, and callus samples were harvested and cut into small pieces. The samples were extracted in 150 mL conical flasks (room temperature) with 25 mL of distilled water and sealed with aluminum foil. On an orbital shaker, the sample mixtures were allowed to stand at room temperature for 1 h in the dark. After 1 h, the samples were filtered through a Whatman filter paper to obtain the aqueous extracts, labelled, and placed in a −80 °C freezer for later analysis [72].

#### 4.5.2. Ferric Reducing Antioxidant Potential Assay (FRAP)

First, 3 mL of FRAP reagent was mixed with 200 µL of each sample extract (10 parts 300 of sodium acetate buffer at pH 3.6, 1 part of 10 TPTZ solution, and 1 part of 20 FeCl_3_.6H_2_O). The reaction mixture was incubated in a 37 °C water bath for 30 min, and the absorbance was measured at 593 nm. The techniques used were adapted versions of Wong et al. [72] and Benzie and Strain [73]. The antioxidant potential was represented as mg Trolox equivalent per gram dry weight (mg TE/g DW) of leaf sample based on the ability to reduce the ferric ions in the extracts.

#### 4.5.3. 2,2-Diphenyl-1-picrylhydrazyl (DPPH) Free Radical Scavenging Assay

The DPPH free radical scavenging assay of each sample was calculated using the method developed by Wong et al. [72] using the free radical DPPH. Methanolic DPPH solution was prepared by dissolving 0.1 mM DPPH in methanol and measuring the absorbance at 515 nm until the absorbance remained stable during the assay cycle. A total of 40 µL leaf extract was combined with 3 mL of 0.1 mM methanolic DPPH solution. The absorbance was measured at 515 nm after 30 min of incubation at room temperature. Total antioxidant content was expressed as mg Trolox equivalent per gram dry weight (mg TE/g DW).

#### 4.5.4. Total Phenolic Content Determination

The total phenolic acid content was measured using the Folin–Ciocalteu phenol reagent method, as described by Singleton and Rossi [74]. One milliliter of each sample extract was applied to a test tube containing 9 mL of sterile water. The mixture was carefully mixed using a vortex machine after 1 mL Folin–Ciocalteu phenol reagent was applied to each test tube. After 5 min of mixing, 10 mL 7% sodium carbonate was added. The overall volume of the mixture was 25 mL after adding 4 mL distilled water. The mixture was then incubated at room temperature for 90 min to complete the reaction. Finally, the absorbance at 750 nm was determined with a spectrophotometer. The total phenolic acid content of the leaf extracts and callus was calculated as mg gallic acid equivalents per gram plant material on a dry basis (mg GAE/g DW).

#### 4.5.5. Total Flavonoid Content Determination

The total flavonoid content was measured using the aluminum chloride colorimetric assay, as described by Marinova et al. [75]. In a test tube, 1 mL of each sample extract was mixed with 4 mL of distilled water. Following this, 0.3 mL 5% sodium nitrite was applied to each test tube. After 5 min, 0.3 mL 10% aluminum chloride was applied to the mixture. At the sixth minute, 2 mL 1 M sodium hydroxide was added. The mixture was then adjusted to 10 mL by adding 2.4 mL distilled water. A vortex machine was used to fully blend the mixture, and the absorbance was determined at 510 nm using a spectrophotometer. The total flavonoid content of the extracts was calculated as mg quercetin equivalents per gram plant material on a dry basis (mg QE/g DW).

### 4.6. Experimental Design and Data Analysis

All experiments were conducted in a completely randomized design and were repeated twice. Each treatment consisted of three replicates. Mean values of various treatments were subjected to analysis of variance (ANOVA), and the significant difference was separated using Duncan’s Multiple Range Test (DMRT). SAS version 9.4 was used to determine the significance at *p* < 0.05.

## 5. Conclusions

Our research successfully identified and compared the biochemical properties of wild plant leaves, leaves from in vitro grown plantlets, and callus of *L. pumila* var. *alata,* and discovered that callus is the best antioxidant source, followed by in vitro plantlets and wild plant. This study adds to the body of knowledge about a more practical, time-saving, and easy-to-cultivate approach for the health supplement and pharmaceutical industries.

## Figures and Tables

**Table 1:** The effects of BAP, kinetin, and zeatin on shoot multiplication from nodal segment of *Labisia pumila* var. *alata* after 6 weeks of culture.

Growth Regulators (mg/L)			No. of Shoots	Length of Shoot (cm)	No. of Leaves
BAP	Kin	Zea			
0	0	0	5.56 abcd	3.89 ab	2.1 bc
1	0	0	6.15 abc	3.55 abc	1.6 bcdef
2	0	0	2.03 g	2.15 efg	1.0 def
3	0	0	3.92 bcdef	2.51 defg	1.73 bcde
4	0	0	4.06 abcdef	2.49 defg	1.4 cdef
5	0	0	3.25 efg	2.37 defg	1.42 cdef
0	0	0	4.19 abcdef	3.19 abcd	1.8 bcd
0	1	0	3.32 efg	2.61 cdefg	0.93 ef
0	2	0	3.13 fg	2.54 defg	0.93 ef
0	3	0	3.61 def	2.26 efg	1.1 def
0	4	0	3.75 cdef	2.71 cdefg	1.4 cdef
0	5	0	2.12 g	2.13 fg	0.8 f
0	0	0	2.74 fg	2.10 g	1.4 cdef
0	0	1	5.36 abcde	4.32 a	2.4 b
0	0	2	6.53 ab	3.50 abc	3.13 a
0	0	3	6.64 a	2.95 bcde	2.27 b
0	0	4	5.29 abcde	2.93 bcdef	1.0 def
0	0	5	6.10 abc	2.89 bcdefg	1.0 def

Means with the same letter in each column are not significantly different at (*p* < 0.05) from each other according to DMRT analysis. BAP = 6-benzylaminopurine, kin = kinetin, and zea = zeatin.

**Table 2:** The effects of 2,4-D and picloram at different concentrations on the callus induction percentage of leaf explants of *Labisia pumila* var. *alata* after 4 weeks of culture.

Growth Regulators (mg/L)		Callus Induction Percentage (%)	Callus Score	Morphology, Texture
2,4-D	Picloram			
0	0	NC	-	NC
0.5	0	60 a	++	Greenish, compact
1	0	6.7 c	++	Greenish, compact
1.5	0	6.7 c	+	Friable, yellowish to greenish
2	0	NC	-	NC
2.5	0	NC	-	NC
0	0.5	6.7 c	+	Friable, yellowish
0	1	50 a	+++	Friable, whitish
0	1.5	30 b	++	Friable, whitish
0	2	27 b	++	Friable, yellowish to whitish
0	2.5	27 b	+	Friable, yellowish to whitish

Means with the same letter are not significantly different at (*p* < 0.05) from each other according to DMRT analysis. NC = no callus, 2,4-D = 2,4-dichlorophenoxyacetic acid, + = callus grown on one edge of leaf, ++ = callus grown on two edges of leaf, +++ = callus grown on three edges of leaf.

**Table 3:** The effects of 0.5 mg/L 2,4-D and 0.25 mg/L different cytokinins on callus induction percentage of leaf explant of *Labisia pumila* var. *alata* after 4 weeks of culture.

Growth Regulators (mg/L)	Callus Formation (%)	Callus Score	Morphology, Texture
0.5 2,4-D + 0.25 zeatin	100 a	+++	Compact, yellowish to greenish
0.5 2,4-D + 0.25 kinetin	73.4 ab	++	Friable, yellowish to greenish
0.5 2,4-D + 0.25 BAP	46.6 b	+	Friable, yellowish to greenish
0.5 2,4-D + 0.25 TDZ	6.6 c	+	Friable, whitish

Means with the same letter are not significantly different at (*p* < 0.05) from each other according to DMRT analysis. BAP = 6-benzylaminopurine, TDZ = thidiazuron, + = callus grown on one edge of leaf, ++ = callus grown on two edges of leaf, +++ = callus grown on three edges of leaf.

**Table 4:** The effects of 1.0 mg/L picloram and 0.5 mg/L different cytokinins on callus induction percentage of leaf explant of *Labisia pumila* var. *alata* after 4 weeks of culture.

Growth Regulators (Mg/L)	Callus Formation Percentage (%)	Callus Score	Morphology, Texture
1.0 picloram + 0.5 zeatin	33.4 ab	+	Greenish, compact
1.0 picloram + 0.5 kinetin	80 a	+++	Whitish, friable
1.0 picloram + 0.5 BAP	66.6 ab	++	Whitish, compact
1.0 picloram + 0.5 TDZ	20 b	+	Whitish to dark brown, compact

Means with the same letter are not significantly different at (*p* < 0.05) from each other according to DMRT analysis. BAP = 6-benzylaminopurine, TDZ = thidiazuron, + = callus grown on one edge of leaf, ++ = callus grown on two edges of leaf, +++ = callus grown on three edges of leaf.

**Table 5:** Total phenolic and flavonoid content of different samples of *Labisia pumila* var. *alata* aqueous extract.

Source of Sample	Total Phenolic Content (mg GAE/g DW)	Total Flavonoid Content (mg QE/g DW)
Wild leaves	1.01 ± 0.07 c	1.67 ± 0.02 c
In vitro plantlets	1.4 ± 0.07 b	1.41 ± 0.68 b
Callus	1.9 ± 0.14 a	2.38 ± 0.18 a

Means with the same letter in each column are not significantly different at (*p* < 0.05) from each other according to DMRT analysis. GAE = gallic acid equivalent, QE = quercetin equivalent.

**Table 6:** Total antioxidant activities using DPPH and FRAP methods of different samples of *Labisia pumila* var. *alata* aqueous extract.

	Total Antioxidant Content (mg TE/g DW)	
Source of Sample	DPPH	FRAP
Wild plant	40.45 ± 2.69 c	81 ± 5.39 c
In vitro plantlet	57.47 ± 2.63 b	115 ± 5.25 b
Callus	75.88 ± 5.80 a	152 ± 11.59 a

Means with the same letter in each column are not significantly different at (*p* < 0.05) from each other according to DMRT analysis. DPPH = 2,2-diphenyl-1-picrylhydrazyl, FRAP = ferric reducing antioxidant potential.

**Table 7:** Pearson correlation of polyphenolic compounds and antioxidant activities of different samples of *Labisia pumila* var. *alata*.

	Variables	1	2	3	4
**1**	TPC	1			
**2**	TFC	0.86 *	1		
**3**	DPPH	0.81 *	0.91 *	1	
**4**	FRAP	0.98 *	0.91 *	0.86 *	1

* is significantly affected at *p* < 0.05. TPC = total phenolic content, TFC = total flavonoid content, DPPH = 2,2-diphenyl-1-picrylhydrazyl, FRAP = ferric reducing antioxidant potential.

## Data Availability

The data presented in this study are available in the article.

## References

[1] Abdullah N., Chermahini S.H., Suan C.L., Sarmidi M.R. (2013). *Labisia pumila*: A review on its traditional, phytochemical and biological uses. World Appl. Sci. J..

[2] Chua L.S., Lee S.Y., Abdullah N., Sarmidi M.R. (2012). Review on *Labisia pumila* (Kacip Fatimah): Bioactive phytochemicals and skin collagen synthesis promoting herb. Fitoterapia.

[3] Ibrahim M.H., Jaafar H.Z. (2011). The relationship of nitrogen and C/N ratio with secondary metabolites levels and antioxidant activities in three varieties of Malaysian Kacip Fatimah (*Labisia pumila* Blume). Molecules.

[4] Ling A.P.K., Tan K.P., Hussein S. (2013). Comparative effects of plant growth regulators on leaf and stem explants of *Labisia pumila* var. *alata*. J. Zhejiang Univ. Sci. B..

[5] Hasan N.A., Hussein S.B., Ibrahim R. Effect of medium pH and sucrose concentrations on adventitious roots induction of Labisia pumila. Proceedings of the IRES 14th International Conference.

[6] Norhaiza M., Maziah M., Hakiman M. (2009). Antioxidative properties of leaf extracts of a popular Malaysian herb, *Labisia pumila*. J. Med. Plants Res..

[7] Pihie L., Hawariah A., Zakaria Z.A., Othman F. (2012). Antiproliferative and proapoptotic effects of *Labisia pumila* ethanol extract and its active fraction in human melanoma HM3KO cells. Evid. Based Compl. Alt. Med..

[8] Karimi E., Jaafar H.Z., Ahmad S. (2013). Antifungal, anti-inflammatory and cytotoxicity activities of three varieties of *Labisia pumila* Benth: From microwave obtained extracts. BMC Compl. Altern. Med..

[9] Ibrahim M.H., Jaafar H.Z.E. (2011). Increased carbon dioxide concentration improves the antioxidative properties of the Malaysian herb, Kacip Fatimah (Labisia pumila Blume). Molecules.

[10] Mohd Hanafi M.M., Yaakob H., Sarmidi M.R., Aziz R., Prieto J.M. (2016). *Marantodes pumilum* L. plant extracts induce apoptosis, cell cycle arrest and inhibit cell migration and invasion on prostate cancer cell lines. Planta Med..

[11] Ahmad S.U., Azam A., Shuid A.N., Mohamed I.N. (2017). Phyto-estrogenic effects of *Marantodes pumilum* (Blume) Kuntze syn. *Labisia pumila* (Blume) Fern.-Vill. for the prevention and treatment of post-menopausal diseases. Indian J. Tradit. Knowl..

[12] Madzuki I.N., Lau S.F., Tantowi N.A.C.A., Ishak N.I.M., Mohamed S. (2018). *Labisia pumila* prevented osteoarthritis cartilage degeneration by attenuating joint inflammation and collagen breakdown in postmenopausal rat model. Inflammopharmacology.

[13] Ibrahim M.H., Jaafar H.Z.E. (2012). Reduced photoinhibition under low irradiance enhanced Kacip Fatimah (*Labisia pumila* Benth) secondary metabolites, phenyl alanine lyase and antioxidant activity. Int. J. Mol. Sci..

[14] Fazwa F., Syafiqah Nabilah S.B., Norhayati S., Norwati M., Marzalina M. (2018). Rapid mass production of elite clone of *Labisia pumila* var. *alata* (KFeFRIM01) for sustainable supply of high-quality planting materials. Int. J. Agric. For. Plant..

[15] Moscatiello R., Baldan B., Navazio L. (2013). Plant cell suspension cultures. Met. Mol. Biol..

[16] Bilušić Vundać V., Pfeifhofer W., Brantner A., Males Z. (2006). Essential oils of seven Stachys taxa from Croatia. Biochem. Syst. Ecol..

[17] Perrino E.V., Valerio F., Gannouchi A., Trani A., Mezzapesa G. (2021). Ecological and plant community implication on essential oils composition in useful wild Officinal species: A pilot case study in Apulia (Italy). Plants.

[18] Karimi E., Jaafar H.Z.E., Ahmad S. (2011). Phytochemical analysis and antimicrobial activities of methanolic extracts of leaf, stem and root from different varieties of *Labisia pumila* Benth. Molecules.

[19] Gupta D. (2015). Methods for determination of antioxidant capacity: A review. Int. J. Pharm. Sci. Res..

[20] Pisoschi A.M., Negulescu G.P. (2011). Methods for total antioxidant activity determination: A review. Biochem. Anal. Biochem..

[21] Stoker H.S. (2013). General, Organic and Biological Chemistry Canada.

[22] Dontha S. (2016). A review on antioxidant methods. Asian J. Pharm. Clin. Res..

[23] Hussein R.A., El-Anssary A.A., Builders P.F. (2018). Plant secondary metabolites: The key drivers of the pharmacological actions of medicinal plants. Herbal Medicine.

[24] Herrmann K.M. (1995). The shikimate pathway as an entry to aromatic secondary metabolism. Plant Physiol..

[25] Mendoza N., Silva E.M.E., Asao T., Asaduzzaman M. (2018). Introduction to phytochemicals: Secondary metabolites from plants with active principles for pharmacological importance. Phytochemicals: Source of Antioxidants and Role in Disease Prevention.

[26] Manach C., Scalbert A., Morand C., Remesy C., Jimenez L. (2004). Polyphenols: Food sources and bioavailability. Am. J. Clin. Nutr..

[27] Pott D.M., Osorio S., Vallarino J.G. (2019). From central to specialized metabolism: An overview of some secondary compounds derived from the primary metabolism for their role in conferring nutritional and organoleptic characteristics to fruit. Front. Plant Sci..

[28] Upadhyay R., Chaurasia J.K., Tiwari K.N., Singh K. (2013). Comparative antioxidant study of stem and stem induced callus of Phyllanthus fraternus Webster—an important antiviral and hepatoprotective plant. Appl. Biochem. Biotechnol..

[29] Nabilah S., Fazwa F., Suhaila S., Norhayati N., Zaki M., Masitah M. (2017). Acclimatization of KFeFRIM01: A superior clone of *Labisia pumila* var. *alata*. Int. J. Environ. Agric. Res..

[30] Marbawi H., Cyril O., David D., Gansau J.A. (2018). In vitro multiple shoot regeneration from stem explant of commercially important medicinal herb *Labisia pumila* var. *pumila*. ASM Sci. J..

[31] Tekdal D., Çetiner S. (2014). The determination of self-compatibility status of *Thermopsis turcica* through histological analysis. J. Appl. Biol. Sci..

[32] Li J.Z., Jing T.Y., Qing G.W. (2017). A protocol for rapid and high-frequency in vitro propagation of *Solanum nigrum* L.. Sains Malays..

[33] Chen H.Y., Liu J., Pan C., Yu J.W., Wang Q.C. (2018). In vitro regeneration of adventitious buds from leaf explants and their subsequent cryopreservation in highbush blueberry. Plant Cell Tissue Organ. Cult..

[34] Nikolić R., Mitić N., Miletić R., Nešković M. (2006). Effects of cytokinins on in vitro seed germination and early seedling morphogenesis in *Lotus corniculatus* L.. J. Plant Growth Regul..

[35] Masekesa T.R., Gasura E., Ngadze E., Icishahayoa D., Kujeke G.T., Chidzwondob F., Robertsona I. (2016). Efficacy of zeatin, kinetin and thidiazuron in induction of adventitious root and shoot from petiole explants of sweet potato cv. Brondal. S. Afr. J. Bot..

[36] Nisha Rani D., Nair G.M. (2006). Effects of plant growth regulators on high frequency shoot multiplication and callus regeneration of an important Indian medicinal plant, Nirgundi (*Vitex negundo* L.). In Vitro Cell. Dev. Biol. Plant.

[37] Sahoo Y., Chand P.K. (1998). Micropropagation of *Vitex negundo* L., a woody aromatic medicinal shrub, through high-frequency axillary shoot proliferation. Plant Cell Rep..

[38] Chandramu C., Rao M., Reddy V.D. (2003). High frequency induction of multiple shoots from nodal explants of *Vitex negundo* L. using sodium sulphate. J. Plant Biotechnol..

[39] Jebakumar M., Jayabalan M. (2000). An efficient method for regeneration of plantlets from nodal explants of *Prosalea corydifolia* Linn. Plant Cell Biotechnol. Mol. Biol..

[40] Husain M.K., Anis M. (2006). Rapid in vitro propagation of *Eclipta alba* (L.) Hassk. through high frequency axillary shoot proliferation. Acta Physiol. Plant..

[41] Raja H.D., Arockiasamy D.I. (2008). In vitro propagation of *Mentha viridis* L. from nodal and shoot tip explants. Plant Tiss. Cult. Biotechnol..

[42] Manivannan A., Soundararajan P., Park Y.G., Jeong B.R. (2015). *In vitro* propagation, phytochemical analysis, and evaluation of free radical scavenging property of Scrophularia kakudensis Franch tissue extracts. BioMed Res. Int..

[43] Varghese T., Rema Shree A.B., Naheesa E., Neelakandan N., Nandakumar S. (2003). In vitro propagation of *Terminalia arjuna* Roxb. multipurpose tree. Plant Cell Biotechnol. Mol. Biol..

[44] Ch B., Rao K., Gandi S., Giri A. (2012). Abiotic elicitation of gymnemic acid in the suspension cultures of Gymnema sylvestre. World J. Microbiol. Biotechnol..

[45] Abdelmageed A.H.A., Faridah Q.Z., Nor Shuhada K., Julia A.A. (2012). Callus induction and plant regeneration of *Michelia champaca* (Magnoliaceae): A multipurpose tree. J. Med. Plants Res..

[46] Ikeuchi M., Sugimoto K., Iwase A. (2013). Plant callus: Mechanisms of induction and repression. Plant Cell.

[47] Cunha A.C.G.D., Ferreira M.F. (1996). Somatic embryogenesis, organogenesis and callus growth kinetics of flax. Plant Cell Tissue Organ Cult..

[48] Sami A.M., Hashish K.I., Sawsan S.S., Lobna S.T. (2016). *In vitro* propagation protocol of Hibiscus syriacus L. plants. Int. J. PharmTech Res..

[49] Oi K., Samuel K., Modeste K.K., Oumar S., Edmond K., Hilaire K.T. (2017). Improved callogenesis and somatic embryogenesis using amino acids and plant growth regulators combination in pineapple [Ananas comosus (L.) Merr (Bromeliaceae)]. Eur. J. Biotechnol. Biosci..

[50] Haida Z., Nakasha J.J., Hakiman M. (2020). In vitro responses of plant growth factors on growth, yield, phenolics content and antioxidant activities of Clinacanthus nutans (Sabah snake grass). Plants.

[51] Zahid N.A., Jaafar H.Z.E., Hakiman M. (2021). Micropropagation of ginger (Zingiber officinale Roscoe) ‘Bentong’ and evaluation of its secondary metabolites and antioxidant activities compared with the conventionally propagated plant. Plants.

[52] Rameshkumar R., Satish L., Pandian S., Rathinapriya P., Rency A.S., Shanmugaraj G., Pandian S.K., Leung D.W., Ramesh M. (2018). Production of squalene with promising antioxidant properties in callus cultures of Nilgirianthus ciliatus. Ind. Crop Prod..

[53] Muthukrishnan S., Kumar T.S., Gangaprasad A., Maggi F., Rao M.V. (2018). Phytochemical analysis, antioxidant and antimicrobial activity of wild and in vitro derived plants of Ceropegia thwaitesii Hook–An endemic species from Western Ghats, India. J. Genet. Eng. Biotechnol..

[54] Hinneburg I., Dorman H.D., Hiltunen R. (2006). Antioxidant activities of extracts from selected culinary herbs and spices. Food Chem..

[55] Kousalya L., Narmatha Bai V. (2016). (2016). Effect of growth regulators on rapid micropropagation and antioxidant activity of Canscora decussata (Roxb.) Roem. & Schult.—A threatened medicinal plant. Asian Pac. J. Reprod..

[56] Song H., Kumar P., Arivazhagan G., Lee S.I., Yoon H.M., Kim I.H., Kwon H.J., Kim J.M., Hakkim F.L. (2012). Antioxidant property of leaves and calluses extracts of *in-vitro* grown 5 different Ocimum species. J. Plant Biotechnol..

[57] Mustapha Z., Harun H. (2015). Phytochemical constituents in leaves and callus of *Ficus deltoidea* Jack var. *kunstleri* (King) Corner. Walailak J. Sci. Technol..

[58] Verpoorte R., Alfermann A.W. (2000). Metabolic Engineering of Plant Secondary Metabolism.

[59] Grąbkowska R., Matkowski A., Grzegorczyk-Karolak I., Wysokińska H. (2016). Callus cultures of Harpagophytum procumbens (Burch.) DC. ex Meisn.; Production of secondary metabolites and antioxidant activity. South Afr. J. Bot..

[60] Abd Samat N.M.A., Ahmad S., Awang Y., Bakar R.A.H., Hakiman M. (2020). Alterations in herbage yield, antioxidant activities, phytochemical contents, and bioactive compounds of sabah snake grass (Clinacanthus nutans L.) with regards to harvesting age and harvesting frequency. Molecules.

[61] Huda-Faujan N., Noriham A., Norrakiah A.S., Babji A.S. (2009). Antioxidant activity of plants methanolic extracts containing phenolic compounds. Afr. J. Biotechnol..

[62] Sun T., Ho C.T. (2005). Antioxidant activities of buckwheat extracts. Food Chem..

[63] Hönig M., Plíhalová L., Husičková A., Nisler J., Doležal K. (2018). Role of cytokinins in senescence, antioxidant defence and photosynthesis. Int. J. Mol. Sci..

[64] Fazal H., Abbasi B.H., Ahmad N., Noureen B., Shah J., Ma D., Chuanliang L., Akbar F., Uddin M.N., Khan H. (2020). Biosynthesis of antioxidative enzymes and polyphenolics content in calli cultures of Prunella vulgaris L. in response to auxins and cytokinins. Artif. Cells Nanomed. Biotechnol..

[65] Piotrowska-Niczyporuk A., Bajguz A. (2014). The effect of natural and synthetic auxins on the growth, metabolite content and antioxidant response of green alga Chlorella vulgaris (Trebouxiophyceae). Plant Growth Regul..

[66] Lukmanul H., Gowri Shankar C., Girija S. (2007). Chemical composition and antioxidant property of holy basil (Ocimum sanctum L.) leaves, stems, and inflorescence and their in vitro callus cultures. J. Agric. Food Chem..

[67] Paula C., Santos G., Rosa M., Seabra P.B., Andrade M., Fernades F. (2002). Phenolic anti-oxidant compounds produced by in vitro shoots of sage (Salvia officinalis L.). Plant Sci..

[68] Barz W., Barz W., Reinhard E., Zenk M.H. (1977). Catabolism of endogenous and exogenous compounds by plant cell cultures. Plant Tissue Culture and Its Biotechnological Application. Proceedings in Life Sciences.

[69] Sargent J.A., Skoog F. (1960). Effects of indoleacetic acid and kinetin on scopoletin and scopolin levels in relation to growth of tobacco tissue in vitro. Plant Physiol..

[70] Skoog F., Montaldi E. (1961). Auxin–kinetin interaction regulating the scopoletin and scopolin levels in tobacco tissue cultures. Proc. Natl. Acad. Sci. USA.

[71] Murashige T., Skoog F. (1962). A revised medium for rapid growth and bio assays with tobacco tissue cultures. Physiol. Plant..

[72] Wong S.P., Lai P.L., Jen H.W.K. (2006). Antioxidant activities of aqueous extracts of selected plants. Food Chem..

[73] Benzie I.F., Strain J.J. (1996). The ferric reducing ability of plasma (FRAP) as a measure of “antioxidant power”: The FRAP assay. Anal. Biochem..

[74] Singleton V.L., Rossi J.A. (1965). Colorimetry of total phenolics with phosphomolybdic-phosphotungstic acid reagents. Am. J. Enol. Viticult..

[75] Marinova D., Ribarova F., Atanassova M. (2005). Total phenolics and total flavonoids in Bulgarian fruits and vegetables. J. Univ. Chem. Technol. Metal..

